# Adenoviral Infections in Bone Marrow Transplanted Adult Patients: A Review of the 44 Cases Reported in the Last 25 Years

**DOI:** 10.7759/cureus.19865

**Published:** 2021-11-24

**Authors:** Stergiani Keramari, Frideriki Poutoglidou, Alexandros Poutoglidis, Damianos Sotiropoulos, Christos Savopoulos, Katerina Chlichlia, Stefanos Chatzis, Angeliki Xagorari, Georgia Kaiafa

**Affiliations:** 1 Department of Paediatrics, University General Hospital of Thessaloniki AHEPA, Thessaloniki, GRC; 2 Department of Clinical Pharmacology, Faculty of Medicine, School of Health Sciences, Aristotle University of Thessaloniki, Thessaloniki, GRC; 3 Department of Otorhinolaryngology-Head and Neck Surgery, “G. Papanikolaou” General Hospital of Thessaloniki, Thessaloniki, GRC; 4 Department of Haematology and Public Cord Blood Bank, “G. Papanikolaou” General Hospital of Thessaloniki, Thessaloniki, GRC; 5 First Propaedeutic Department of Internal Medicine, University General Hospital of Thessaloniki AHEPA, Thessaloniki, GRC; 6 Department of Molecular Biology and Genetics, Democritus University of Thrace, Alexandroupolis, GRC; 7 Department of Surgery, 251 Airforce Hospital, Athens, GRC

**Keywords:** bone marrow transplantation, leukemia, antiviral therapy, infection, adenovirus

## Abstract

Adenoviral infections in immunocompromised individuals may be life-threatening conditions. The aim of this review is to document all the reported cases of adenoviral infection is patients having undergone bone marrow transplantation (BMT). A comprehensive literature search of the databases Pubmed, Science Direct, and Google Scholar was conducted to identify all the case reports of adenoviral infections in BMT patients. A total of 30 articles with 44 patients were included. The most common underlying condition was acute lymphocytic leukemia (23%) followed by acute myeloid leukemia (18%). The most common site of infection was disseminated (50%), followed by liver infection (8%) and hemorrhagic cystitis (8%). Cidofovir was administered in 40.9% of the cases, and death was reported in 34.4% of them. Ribavirin was administered as monotherapy in 15.9% of patients, with a mortality rate of 57.1%. We found that the antiviral drug option had no statistically significant effect on the mortality rate (p=0.242). Also, the absence of graft-versus-host disease (GVHD) was not associated with an improved outcome (p=0.523). There was, however, a statistically significant difference in the outcome based on the site of infection (p=0.005), with a higher rate of mortality in the disseminated and gastrointestinal cases. To the best of our knowledge, this is the first review documenting all the cases of adenoviral infections in BMT patients. Future randomized studies are needed to validate the results of the present study.

## Introduction and background

Adenoviruses are common pathogens that cause a wide range of illnesses and symptoms. They are extremely widespread and their seropositivity among children younger than four years old has been reported as high as 80% [1-3]. In the vast majority of immunocompetent hosts, adenoviral infections are self-limited and fatal cases are rare [4-5]. However, adenoviral infection in immunocompromised individuals, including patients having undergone bone marrow transplantation (BMT), maybe a life-threatening condition.

The most common sites of adenoviral infections include the respiratory tract, gastrointestinal tract, and urinary tract. Disseminated adenoviral infections can also occur, mainly in stem cells transplanted recipients and rarely in healthy hosts, and are associated with high mortality rates [2,6]. The diagnosis of a disseminated adenoviral infection is established when two or more sites are affected or when the virus is isolated from the blood. In some studies, moderate to severe graft-versus-host disease (GVHD) has been referred to as a risk factor for severe manifestations of adenoviral infections in BMT patients [6-10]. The efficacy of antiviral therapeutic strategies against adenoviral infection is still debatable and no specific therapy is strongly recommended.

The aim of the present review is to document all the reported cases of adenoviral infections in BMT patients. Data including the underlying hematologic disorder, the site of infection, the presence or absence of GVHD, the antiviral drug treatment, and the outcome of the cases are also recorded and statistically analyzed.

## Review

Methods

We performed a comprehensive electronic search of the PubMed, Science Direct, and Google Scholar electronic databases to identify all the reported cases of adenoviral infections in BMT patients from their inception up to October 2021. The search terms used were as follows: "Bone Marrow Transplantation", "Adenovirus", "Adenoviral Infection". Exclusion criteria were: (1) reports of pediatric population, (2) the presence of HIV infection, (3) other causes of immunodeficiency. Conference abstracts were not included. Any duplicates or obviously irrelevant studies were also excluded.

A dedicated data extraction form was developed for recording all relevant details, involving publication details (author(s) and year of publication), number of patients, patients' age, underlying hematologic condition, site of infection, the presence or absence of GVHF, the antiviral drug option, and the outcome. 

The data were entered into an Excel sheet (Microsoft Corporation, Redmond, WA) and were analyzed using the Statistical Program for Social Sciences (SPSS) 21.0 (IBM Corporation, Armonk, NY). The graphs were created using GraphPad Prism software version 9.0 (GraphPad Software, La Jolla, CA). Categorical variables were expressed as frequencies and percentages. Continuous variables were given as mean (range). The data were analyzed using contingency tables. The chi-square test of independence was used to determine whether there was an association between the categorical variables. A p-value of less than 0.05 was considered statistically significant.

Taking into account that this study is a review of published articles, neither ethics approval nor informed consent was needed.

Results

A total of 30 articles, with 44 patients, published from 1996 to 2020 were included (Table 1) [11-40]. The mean age of the patients was 42.89 (14.24) years and 29 of them (66%) were men. Acute lymphocytic leukemia was the most common underlying condition (10/44, 23%), followed by acute myeloid leukemia (8/44, 18%), chronic myeloid leukemia (6/44, 14%), and non-Hodgkin lymphoma (5/44, 11%) (5/44). There was one case for each of the following diseases: myelodysplastic syndrome, aplastic anemia, Hodgkin lymphoma, chronic lymphocytic leukemia, and Waldenstrom’s macroglobulinemia. The underlying condition was not mentioned in five of the patients (11%).

**Table 1:** Reported cases of adenoviral infection in bone marrow transplanted patients M: male, F: female, ADV: adenoviral, ALL: acute lymphocytic leukemia, AML: acute myeloid leukemia, CML: chronic myeloid leukemia, NHL: non-Hodgkin lymphoma, MDS: myelodysplastic syndrome, HD: Hodgkin lymphoma, CLL: chronic lymphocytic leukemia, N.A.: not available

AUTHOR	YEAR	AGE/GENDER	DISEASE	ADV INFECTION	GVHD	ANTIVIRAL TREATMENT	OUTCOME
Bertheau P. et al [11]	1996	22(M)	CML	Liver infection	YES	IVIG	DEATH
Lakhani A. et al [12]	1999	28(F)	CML	Cystitis	YES	Ribavirin	IMPROVEMENT
Hale G.A. et al [13]	1999	24(F)	ALL	Disseminated	NO	N.A.	DEATH
Chakrabarti S. et al [14]	1999	44(M)	CML	Disseminated	YES	Ribavirin	DEATH
Somervaille TC et al [15]	1999	39(F)	HD	Liver infection	N.A.	NO	DEATH
Fassas A.B. et al [16]	2000	62(M)	NHL	Disseminated	YES	N.A.	DEATH
Chakrabarti S. et al [17]	2002	43(M)	N.A.	Liver infection	YES	Ribavirin	DEATH
Chakrabarti S. et al [17]	2002	22(F)	N.A.	Disseminated	NO	Ribavirin	DEATH
Wang W.H. et al [18]	2003	21 (M)	ALL	Liver infection	YES	N.A.	DEATH
Abe S. et al [19]	2003	41(M)	ALL	Cystitis	YES	Ribavirin	IMPROVEMENT
Avivi I. et al [20]	2004	22(F)	N.A.	Disseminated	NO	Ribavirin	DEATH
Avivi I. et al [20]	2004	38(M)	N.A.	Liver infection	YES	NO	DEATH
Avivi I. et al [20]	2004	44(F)	N.A.	Liver infection	YES	NO	DEATH
Fanourgiakis P. et al [21]	2005	34 (M)	CML	Cystitis	YES	Cidofovir	DEATH
Nakazawa H. et al [22]	2006	51(F)	ALL	Disseminated	YES	NO	DEATH
Castleton A. et.al [23]	2007	44(M)	AML	Keratoconjunctivitis	NO	Cidofovir	IMPROVEMENT
Neofytos D. et al [24]	2007	23(N.A.)	CML	Liver infection	NO	Cidofovir +IVIG	IMPROVEMENT
Neofytos D. et al [24]	2007	39(M)	ALL	Colitis	NO	Cidofovir +IVIG	DEATH
Neofytos D. et al [24]	2007	43(M)	AML	Disseminated	N.A.	Cidofovir +IVIG	IMPROVEMENT
Neofytos D. et al [24]	2007	73(M)	AML	Colitis	YES	Cidofovir +IVIG	DEATH
Kalpoe J.S. et al [25]	2007	60(M)	CLL	Disseminated	N.A.	Cidofovir	DEATH
Willems L. et. al [26]	2008	26(M)	ALL	Disseminated	YES	Cidofovir	IMPROVEMENT
Suzuki H.I. et al [27]	2008	35(F)	MDS	Disseminated	NO	Ganciclovir + IVIG	IMPROVEMENT
Forstmeyer D. et al [28]	2008	39(M)	NHL	Disseminated	NO	Cidofovir +IVIG +Ribavirin	DEATH
Fowler C.J. et al [29]	2010	67(M)	NHL	Liver infection	YES	NO	DEATH
Fowler C.J. et al [29]	2010	44(M)	NHL	Disseminated	YES	Cidofovir +IVIG	IMPROVEMENT
Fowler C.J. et al [29]	2010	39(M)	CML	Disseminated	YES	Cidofovir	DEATH
Fowler C.J. et al [29]	2010	57(F)	NHL	Disseminated	YES	Cidofovir +IVIG	IMPROVEMENT
Fowler C.J. et al [29]	2010	42(F)	ALL	Disseminated	YES	Cidofovir	DEATH
Fowler C.J. et al [29]	2010	49(M)	Multiple Myeloma	Disseminated	YES	Cidofovir +IVIG	DEATH
Inoue N. et al [30]	2010	45(M)	AML	Disseminated	NO	N.A.	IMPROVEMENT
Alfson E.D. et al [31]	2013	58(F)	ALL	CNS	N.A.	N.A.	DEATH
Awosika O. et al [32]	2013	58(F)	ALL	CNS	N.A.	Cidofovir	DEATH
Mochizuki K. et al [33]	2014	50(M)	Multiple Myeloma	Disseminated	NO	Ganciclovir	DEATH
Mochizuki K. et al [33]	2014	41 (F)	AML	Disseminated	YES	Ganciclovir	DEATH
Sahu K.K. et al [34]	2015	36(M)	AML	Cystitis	YES	Ribavirin	IMPROVEMENT
Detrait M. et al [35]	2015	27(F)	AML	Disseminated	NO	Valaganciclovir	DEATH
Keyes A. et al [36]	2016	20(F)	Aplastic Anemia	Disseminated	NO	Cidofovir +IVIG +Ribavirin	DEATH
Sakurada N. et al [37]	2016	63(M)	AML	Cystitis	YES	N.A.	IMPROVEMENT
Sakurada N. et al [37]	2016	63(M)	Waldenstrom's Macroglobulinemia	Cystitis	NO	IVIG	IMPROVEMENT
Sakurada N. et al [37]	2016	40(M)	ALL	Cystitis	YES	Cidofovir +IVIG	IMPROVEMENT
Claveau J.S. et al [38]	2017	60(M)	Multiple Myeloma	CNS	YES	NO	DEATH
Shimizu T. et al [39]	2019	45(M)	MDS	Cystitis	NO	Cidofovir	IMPROVEMENT
Yasuda et al [40]	2019	66(M)	Multiple Myeloma	Disseminated	N.A.	Ganciclovir	DEATH

The most common adenoviral infection was the disseminated one, in 22 of the patients (50%), followed by adenoviral liver infection and adenoviral hemorrhagic cystitis (8/44, 18% for both of them), central nervous system adenoviral infection (3/44, 7%), adenoviral colitis (2/44, 5%), and adenoviral hemorrhagic keratoconjunctivitis (1/44, 2%). GVHD was present in 24 cases (55%). The presence or absence of GVHD was not mentioned in five patients (11%).

**Figure 1:**
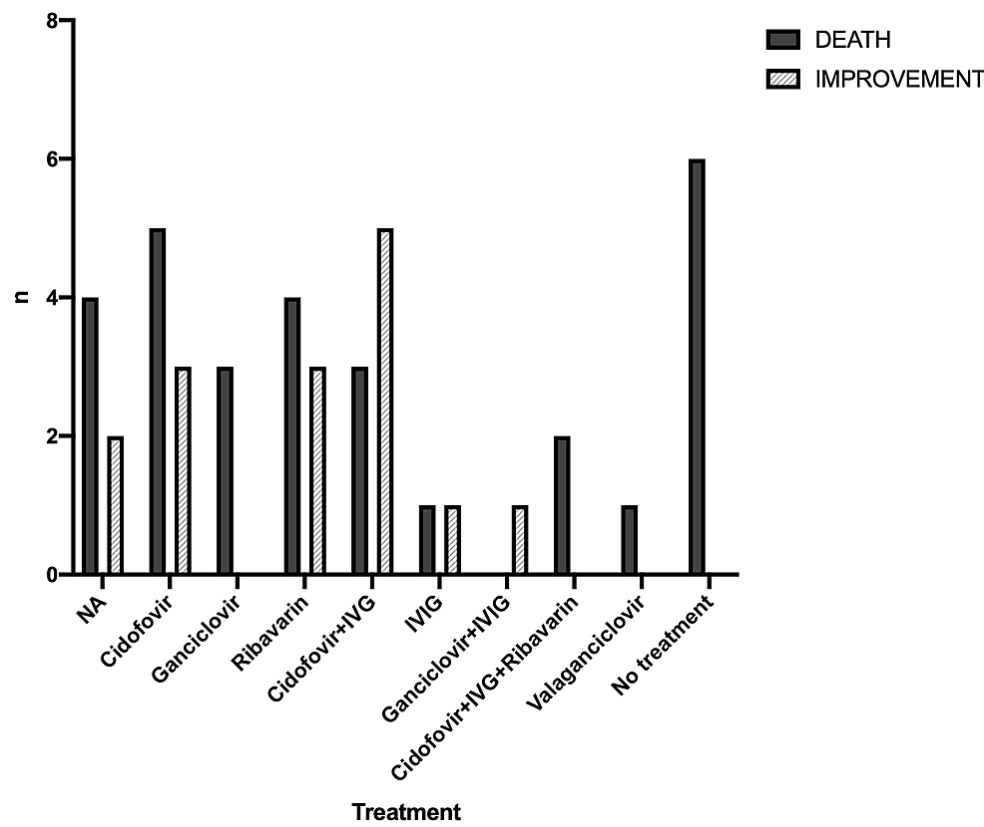
Outcomes in relation to the antiviral treatment used NA: not available, IVIG: intravenous immunoglobulin

The most common antiviral drug used was cidofovir in 18 of the cases (40.9%), either as monotherapy (8/44) or combined with intravenous immunoglobulin (IVIG) (10/44) and death was reported in 34.4% of them. In particular, there was a 62.8% mortality rate in the monotherapy group and a 37.5% mortality rate in the combined treatment group. Other treatment options included ribavirin (7/44, 15.9%), ganciclovir (4/44, 9%), and IVIG administered intravenously (2/44, 4.5%). The mortality rate in the patients treated with ribavirin was 57.1% and in patients treated with ganciclovir 25%. There was no statistically significant difference in the outcome with respect to the antiviral treatment used (x^2^=11.52, df=9, p=0.242) (Figure 1). There were six patients who received no antiviral treatment and the outcome was death for all of them. In six patients, the therapeutic strategy was not mentioned.

Regarding the site of infection, the highest death rate was in patients with adenoviral liver infection (7/8, 88%), followed by patients with disseminated infection (16/22, 73%). The lowest mortality rate was observed in patients with adenoviral hemorrhagic cystitis (1/8, 13%). The two patients who reported gastrointestinal tract and CNS infections both died while the one patient with keratoconjunctivitis improved.

**Figure 2:**
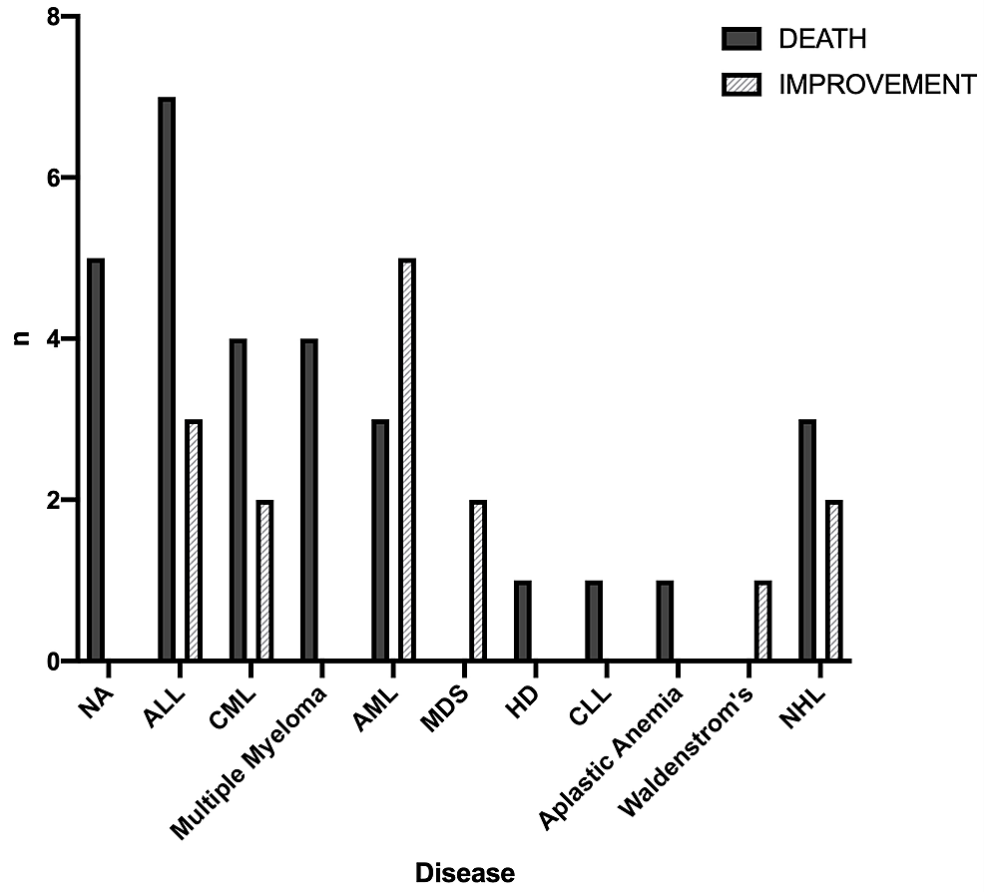
Outcomes in relation to the underlying hematologic condition NA: not available, ALL: acute lymphocytic leukemia, CML: chronic myeloid leukemia, AML: acute myeloid leukemia, MDS: myelodysplastic syndrome, HD: Hodgkin lymphoma, CLL: chronic lymphocytic leukemia, NHL: non-Hodgkin lymphoma

**Figure 3:**
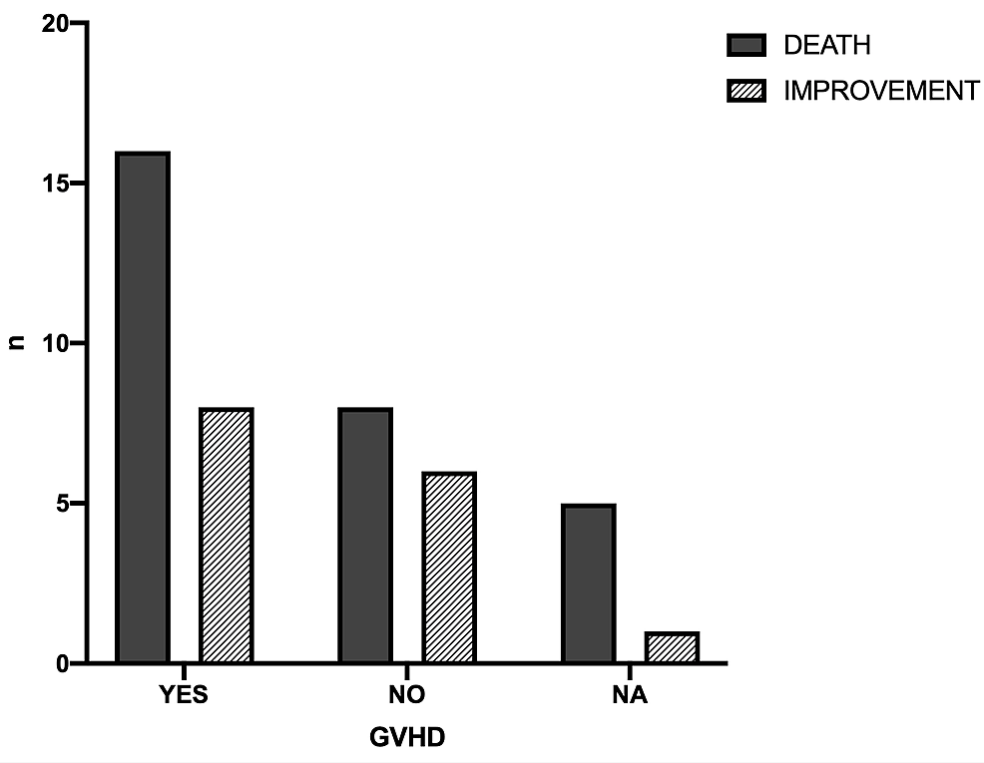
Outcomes in relation to the presence or absence of graft-versus-host disease GVHD: graft-versus-host disease, NA: not available

**Figure 4:**
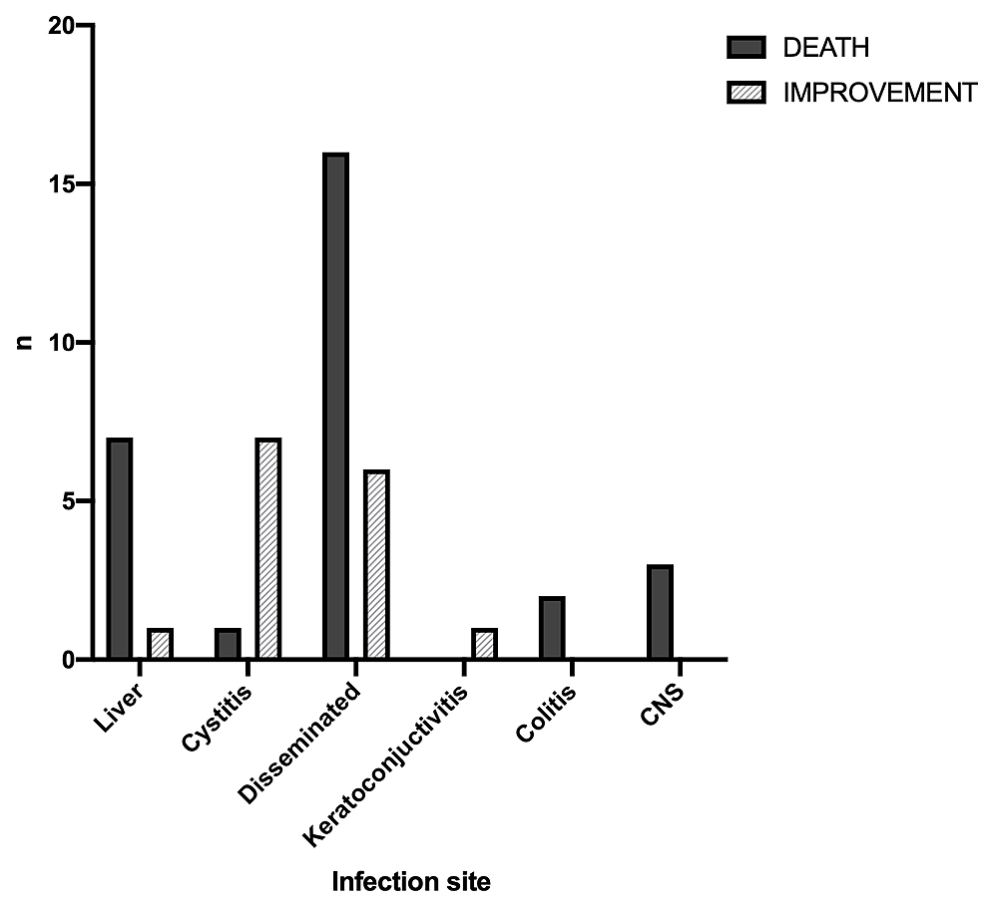
Outcomes in relation to the site of the adenoviral infection CNS: central nervous system

The total mortality rate in BMT patients with adenoviral infection was 65.9% (29/44). Neither the underlying hematologic disorder (Figure 2) nor the presence of GVHD (Figure 3) had a significant effect on the mortality rate (x^2^=15.03, df=10, p=0.131 and x^2^=1.3, df=2, p=0.523, respectively). However, there was a statistically significant difference in the outcome between the different sites of infection (x^2^=16.79, df=5, p=0.005) (Figure 4).

Discussion

Adenoviruses are nonenveloped, double-stranded DNA viruses of the Adenoviridae family, which are extremely common pathogens. Infection by an adenovirus has a wide range of manifestations that mainly depend on the immunologic status of the patient and may range from asymptomatic infection to a severe life-threatening condition.

Adenoviruses may infect a variety of systems such as the respiratory, gastrointestinal, or urinary tract. Adenoviral infections account for 1% to 7% of respiratory tract infections, and typical symptoms include fever, pharyngitis, tonsillitis, cough, and a sore throat. Patients with adenoviral gastrointestinal tract infections usually present with symptoms such as gastroenteritis, vomiting, and diarrhea. Adenoviral urinary tract infections typically cause symptoms such as dysuria and hematuria, which might rarely lead to hemorrhagic cystitis. Finally, ocular adenoviral infection manifestations include epidemic keratoconjunctivitis, pharyngoconjunctival, fever, and nonspecific conjunctivitis. Nosocomial transmissions via environmental contamination, like ophthalmic instruments or eyedrops, have also been reported [41-43].

Adenoviral infections in immunocompromised individuals are serious conditions and their fatality rates may be as high as 60% to 80%. Disseminated infections are common among immunocompromised patients and multiorgan failure is a well-known complication. Persistent adenoviral infections can be manifested as pneumonia, renal failure, hepatitis, hemorrhagic cystitis, or encephalitis. In BMT patients, adenoviral infection is the third most common viral infection after human herpes simplex virus and cytomegalovirus [6-7]. Adenoviral infections that occur within the first 30 days to 90 days after transplantation are often a result of recent exposure while adenoviral infections that occur within 90 days or more are usually a result of adenoviral reactivation [8-9]. It has been reported that moderate to severe GVHD is a risk factor for severe manifestations of adenoviral infections in BMT patients [6-10]. Other risk factors reported are severe T-cell depletion and human leukocyte antigen (HLA) mismatch [44-48].

Immunocompetent hosts usually do not require specific therapy, and they produce neutralizing antiviral antibodies within two weeks from the infection. This, however, does not apply to immunocompromised patients. Unfortunately, there are no specific guidelines for the treatment of adenoviral infections in BMT patients. Treatment options include various antiviral drugs, such as ribavirin and cidofovir, or immunoglobulin. Those drugs have been shown to reduce the viral load and are associated with improved outcomes.

This is a review of 44 reported cases of adenoviral infections in adult patients with hematological diseases having undergone BMT. The present study indicated that the prognosis of adenoviral infections in BMT patients does not correlate with the underlying hematologic disorder or the antiviral treatment used. The prognosis, however, is poor in patients that do not receive any antiviral treatment at all. We found that the site of infection is the most important prognostic factor in those infections. Disseminated and hepatic forms are associated with the worst prognosis.

The present study had some limitations. The evidence obtained comes only from case reports. Future prospective randomized studies are needed to validate the results of the present study. There is a limited number of cases reported in the literature, and the absence of statistical significance may be related to this. Finally, in many of the studies, the presence or absence of GVHD and the antiviral treatment used are not reported, and this could have altered the results. Unfortunately, despite our efforts, we were not able to retrieve this information.

## Conclusions

Adenoviral infections are extremely common and with a wide spectrum of clinical manifestations. The present study is a review of all the cases of adenoviral infections in BMT patients reported in the literature. To the best of our knowledge, this is the first study documenting these cases and analyzing them in order to draw conclusions.

Based on our findings, adenoviral infections represent serious conditions in patients having undergone BMT and are associated with high mortality rates. The disseminated and hepatic infections are the most serious and with the worst outcomes. However, we found no correlation between the type of underlying condition or the presence of GVHD and mortality rates. There are no formal guidelines regarding the treatment of adenoviral infections in BMT patients, however, cidofovir and ribavirin are the most commonly employed by clinicians. We found no statistically significant differences with respect to the antiviral treatment used, but this could be related to the limited number of cases. It should be emphasized that the patients that did not receive any antiviral treatment had the worst outcomes. Taking into account the severity of those infections in this group of patients and the lack of formal guidelines for their treatment, future prospective randomized studies are necessary.
